# PPP1R18-mediated activation of Wnt/β-catenin and EMT is dependent on ERK signaling in clear cell renal cell carcinoma

**DOI:** 10.1186/s12935-026-04266-7

**Published:** 2026-05-10

**Authors:** Xing Ji, Yongyang Yun, Zhenpeng Zhu, Tianyu Wu, Mingjian Ruan, Yu Fan, Qian Zhang

**Affiliations:** 1 Department of Urology, Peking University First Hospital, Beijing, 100034 China; 2 Institution of Urology, Peking University, Beijing, 100034 China; 3 National Urological Cancer Center, Beijing, 100034 China; 4 Beijing Key Laboratory of Urogenital Diseases (Male) Molecular Diagnosis and Treatment Center, Beijing, 100034 China; 5 Department of Urology, Beijing Shijitan Hospital, Capital Medical University, Beijing, 100038 China

**Keywords:** PPP1R18, Clear cell renal cell carcinoma, ERK, Wnt/β-catenin, Epithelial-mesenchymal transition, Prognosis

## Abstract

**Background:**

Advanced clear cell renal cell carcinoma (ccRCC) carries a dismal prognosis, urging the discovery of novel therapeutic targets. Here, we investigate the uncharacterized protein PPP1R18 as a potential driver of ccRCC malignancy.

**Methods:**

PPP1R18 expression and its prognostic value were evaluated using TCGA, GEO, and CPTAC databases, with validation in clinical samples and cell lines. The biological functions of PPP1R18 were assessed in vitro using siRNA-mediated knockdown in a series of in vitro assays and an in vivo xenograft model. The underlying molecular mechanisms were explored through bioinformatic analysis, Western blotting, and rescue experiments using specific pathway agonists and inhibitors to delineate the signaling cascade.

**Results:**

PPP1R18 is significantly upregulated in ccRCC and serves as a robust independent predictor of poor survival. Knockdown of PPP1R18 substantially impaired ccRCC cell proliferation, migration, and invasion in vitro and suppressed tumor growth in vivo. Mechanistically, PPP1R18 depletion suppressed the phosphorylation of ERK and inhibited the Wnt/β-catenin pathway, leading to a reversal of the epithelial-mesenchymal transition (EMT) phenotype. Notably, activation of the ERK pathway rescued the inhibitory effects of PPP1R18 knockdown on Wnt/β-catenin signaling and malignant phenotypes. This rescue was subsequently abrogated by a Wnt/β-catenin inhibitor, confirming that Wnt/β-catenin acts downstream of the PPP1R18/ERK axis.

**Conclusion:**

PPP1R18 is a crucial oncogenic driver and prognostic biomarker in ccRCC. It promotes malignant phenotypes by activating the novel ERK/Wnt/β-catenin signaling axis, offering a potential new target for ccRCC treatment.

**Graphical abstract:**

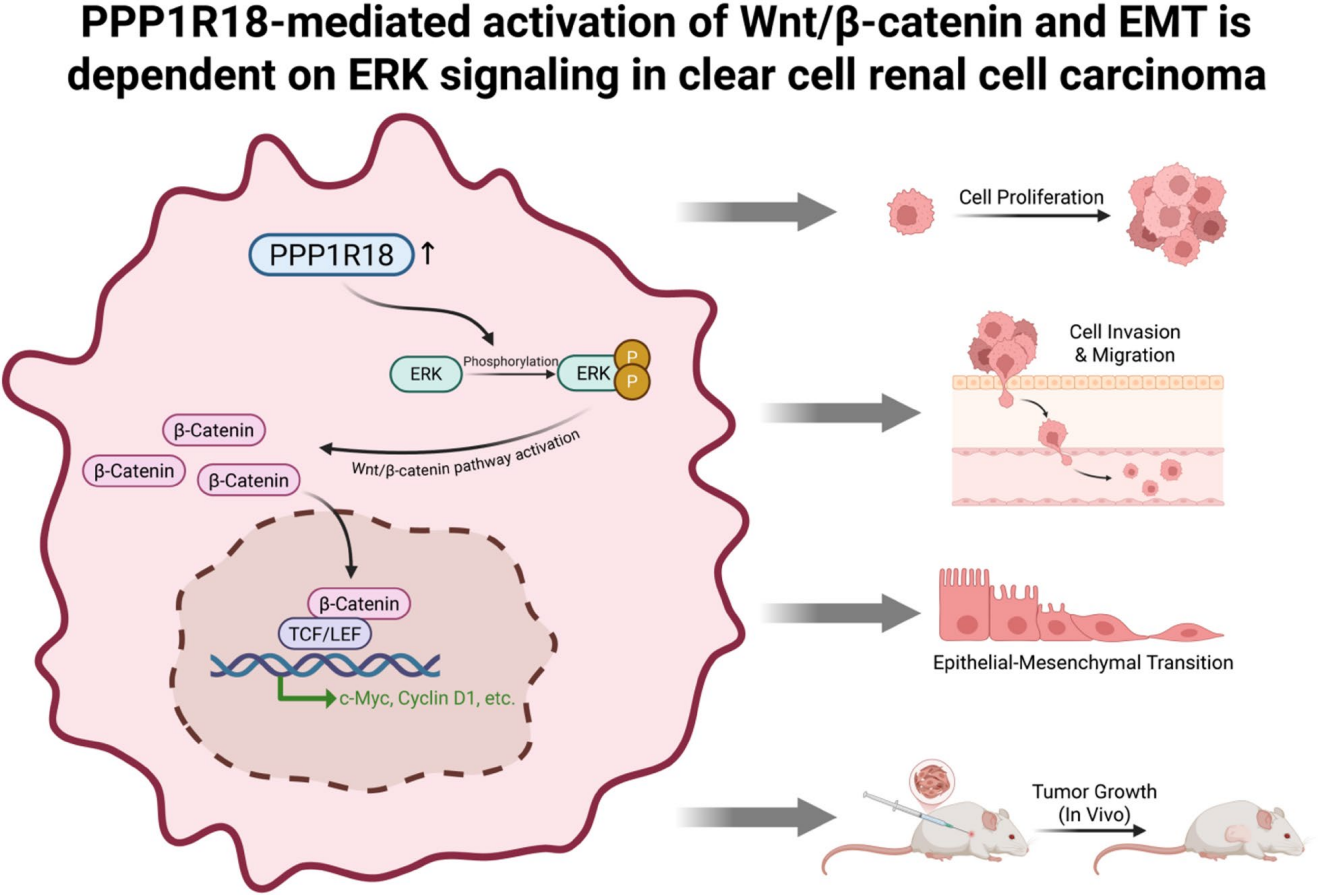

**Supplementary Information:**

The online version contains supplementary material available at 10.1186/s12935-026-04266-7.

## Introduction

 Renal cell carcinoma (RCC) is one of the most common malignancies of the urinary system. Owing to the widespread adoption of diagnostic imaging, the global incidence of RCC has been steadily increasing, with over 430,000 new cases reported in 2020 [1–6]. Clear cell renal cell carcinoma (ccRCC) is the predominant pathological subtype, accounting for approximately 75–80% of all RCC cases [7]. While surgical resection offers a favorable prognosis for localized ccRCC, the treatment of advanced or metastatic disease remains challenging [8]. Approximately one-third of patients experience recurrence after radical surgery [8–10]. Although immune checkpoint inhibitors (ICIs) have significantly improved outcomes for advanced RCC patients, their efficacy is often constrained by primary or acquired resistance and the risk of severe immune-related adverse events [11–17]. Therefore, there is an urgent need to elucidate the molecular mechanisms underlying ccRCC and identify novel therapeutic targets.

Protein phosphatase 1 regulatory subunit 18 (PPP1R18) is a crucial regulatory subunit of protein phosphatase 1 (PP1) [18–20]. By docking with the PP1 catalytic subunit, PPP1R18 targets PP1 to the F-actin cytoskeleton, thereby activating downstream signaling pathways; it also functions as an actin-binding protein. Recently, the role of PPP1R18 in tumorigenesis and progression has garnered increasing interest. Studies have demonstrated that PPP1R18 is significantly upregulated in esophageal squamous cell carcinoma, correlating with tumor invasiveness and poorer prognosis via the calcineurin-mediated ERK pathway [21]. Although a few studies have indicated an association between high PPP1R18 expression and adverse outcomes in ccRCC, the underlying molecular mechanisms of PPP1R18 in ccRCC remain largely unexplored [22, 23].

This study aims to investigate the role of PPP1R18 in ccRCC progression. By elucidating its oncogenic mechanisms in ccRCC, we seek to provide a theoretical foundation and experimental evidence for developing novel targeted therapies and strategies against this malignancy.

## Materials and methods

### Bioinformatic analysis

Transcriptome profiles and corresponding clinical information for clear cell renal cell carcinoma (ccRCC) were obtained from The Cancer Genome Atlas Kidney Renal Clear Cell Carcinoma project (TCGA-KIRC; https://portal.gdc.cancer.gov/projects/TCGA-KIRC), the Gene Expression Omnibus (GEO; https://www.ncbi.nlm.nih.gov/geo/), and the UALCAN database (http://ualcan.path.uab.edu/analysis-prot.html). The TCGA-KIRC dataset included 539 ccRCC and 72 normal tissue samples. The GEO datasets GSE167093 and GSE40435 contained 254 and 101 pairs of ccRCC and adjacent non-tumorous tissue samples, respectively. The UALCAN portal provided proteomic data from the Clinical Proteomic Tumor Analysis Consortium (CPTAC), comprising 219 ccRCC and 169 normal samples. All public data analyses were conducted using R software (version 4.3.1, R Foundation for Statistical Computing, Vienna, Austria). Differential expression and survival analyses were performed using the stats, car, and survival packages.

To investigate the molecular mechanisms of PPP1R18 in ccRCC, enrichment analyses were performed. First, differentially expressed genes (DEGs) between PPP1R18-high and PPP1R18-low expression groups within the TCGA-KIRC dataset were identified using the DESeq2 package. DEGs with a log2(FoldChange) > 0.585 and an adjusted p-value (p.adj) < 0.05 were selected for subsequent Gene Ontology (GO) and Kyoto Encyclopedia of Genes and Genomes (KEGG) pathway enrichment analyses. Gene Set Enrichment Analysis (GSEA) was conducted on all DEGs (p.adj < 0.05) using the Hallmarks gene set (h.all.v7.5.1.symbols.gmt) as the reference [24]. Pathways with a false discovery rate (FDR) < 0.25 and a p.adj < 0.05 were considered significantly enriched. All bioinformatic results were visualized using the ggplot2 package.

### Patient specimens

This study included 20 pairs of liquid nitrogen-frozen ccRCC and matched adjacent non-tumorous tissues. All specimens were collected from patients who underwent nephrectomy at the Department of Urology, Peking University First Hospital, between 2022 and 2023. Pathological diagnoses for all samples were made according to the World Health Organization (WHO) classification guidelines and confirmed by at least two board-certified uropathologists. The study was approved by the Ethics Committee of Peking University First Hospital (2024Yan658-001), and written informed consent was obtained from all participating patients.

### Cell culture and siRNA transfection

The human ccRCC cell lines 786-O, Caki-1, and OSRC-2, and the normal renal cell lines HEK-293 and HK-2 were purchased from the American Type Culture Collection (ATCC, Manassas, VA, USA). All cells were maintained in DMEM (Gibco, Grand Island, NY, USA) supplemented with 10% fetal bovine serum (FBS; Invitrogen, Carlsbad, CA, USA) in a humidified incubator at 37 °C with 5% CO₂.

PPP1R18 was knocked down in ccRCC cells using small interfering RNA (siRNA). The siRNA sequences were as follows: si-PPP1R18 (5′-GAGAAGAAAGACAAGACUACU-3′) and a negative control, si-NC (5′-CCUACGCCACCAAUUUCGU-3′), which were synthesized by Tianyi Huiyuan Co. (Beijing, China). Transfection was performed using Lipofectamine RNAiMAX (Thermo Fisher Scientific, Waltham, MA, USA) according to the manufacturer’s protocol. Briefly, cells were seeded in 6-well plates and grown to approximately 70% confluence. For each well, 30 pmol of siRNA and 9 µL of Lipofectamine RNAiMAX were diluted in 150 µL of Opti-MEM (Gibco), respectively. The two solutions were then combined, incubated at room temperature for 5 min, and added dropwise to the cells. After 72 h of incubation, the knockdown efficiency was assessed by Western blotting.

### Plasmid construction and transfection

To generate an siRNA-resistant PPP1R18 (PPP1R18^si−res^), the coding sequence was recoded with synonymous substitutions within the siRNA recognition site, producing a modified segment (5′-GCGAGGAGAGGCAGGATTATT-3′) that preserved the native amino acid sequence. The recoded PPP1R18 open reading frame was synthesized and subcloned into the pcDNA3.1 vector under a CMV promoter. For rescue assays, ccRCC cells were initially transfected with si-PPP1R18 using Lipofectamine RNAiMAX, followed 24 h later by transfection with PPP1R18^si−res^ using Lipofectamine 3000 according to the manufacturer’s protocol. Cells were collected 72 h post-transfection for functional assays or Western blot analysis.

### Western blotting

Total and nuclear proteins were extracted using a Total Protein Extraction Kit (KeyGEN BioTECH, KGB5303) and a Nuclear Protein Extraction Kit (Beyotime, P0027), respectively, following the manufacturers’ instructions. Protein concentration was quantified using the BCA method. An aliquot of 20 µg of protein from each sample was separated by SDS-PAGE and transferred to a polyvinylidene fluoride (PVDF) membrane via wet transfer. The membranes were blocked with 5% non-fat milk and then incubated with primary and secondary antibodies. Protein bands were visualized using Pierce™ ECL Western Blotting Substrate (Thermo Fisher Scientific) and detected with a ChemiDoc Imaging System (Bio-Rad, Hercules, CA, USA). The primary antibody used were anti-PPP1R18 (Santa Cruz Biotechnology, sc-376816, 1:500), anti-GAPDH (Proteintech, 10494-1-AP, 1:10000), anti-Beta Actin (Proteintech, 66009-1-Ig, 1:10000), anti-Histone H3 (Proteintech, 17168-1-AP, 1:5000), anti-N-cadherin (Proteintech, 22018-1-AP, 1:5000), anti-E-cadherin (Proteintech, 20874-1-AP, 1:5000), anti-Vimentin (Proteintech, 10366-1-AP, 1:20000), anti-Phospho-ERK1/2 (Proteintech, 28733-1-AP, 1:2000), anti-ERK1/2 (Proteintech, 11257-1-AP, 1:5000), anti-β-Catenin (CST, 8480, 1:1000), anti-Cyclin D1 (Proteintech, 60186-1-Ig, 1:10000), anti-c-Myc (CST, 5605, 1:1000).

### Cell counting kit-8 (CCK-8) assay

Cell viability was assessed using a CCK-8 kit (Dojindo, Kumamoto, Japan). Transfected 786-O and Caki-1 cells were seeded into 96-well plates at a density of 800 cells per well. At 0, 24, 48, 72, and 96 h post-seeding, 100 µL of DMEM medium containing 10% CCK-8 was added to each well. After a 2-hour incubation period, the absorbance at 450 nm was measured using a microplate reader.

### Wound healing assay

Cell migration was evaluated using a wound healing assay. Transfected 786-O and Caki-1 cells were grown to > 90% confluence in 6-well plates. A scratch was created in the cell monolayer using a sterile 200 µL pipette tip. After removing cell debris by washing with PBS, the cells were cultured in serum-free medium. Images of the wound were captured at 0 h and every 6 h thereafter using an inverted microscope (Olympus, Tokyo, Japan) at 20× magnification. The wound closure area was quantified using the “Wound Healing Size Tool” plugin in ImageJ software [25].

### Transwell assay

Cell migration and invasion were assessed using Transwell chambers (Corning, Corning, NY, USA). For invasion assays, the upper chambers were pre-coated with Matrigel; for migration assays, uncoated chambers were used. Transfected 786-O (1 × 10⁴ cells/well) and Caki-1 (5 × 10⁴ cells/well) cells were resuspended in serum-free DMEM and seeded into the upper chambers. The lower chambers were filled with 600 µL of DMEM containing 10% FBS. After 24 h of incubation, cells that had migrated or invaded to the lower surface of the membrane were fixed with 4% paraformaldehyde, non-migrated cells on the upper surface were removed with a cotton swab, and the migrated/invaded cells were stained with 0.1% crystal violet. Images were captured using an inverted microscope (Olympus) at 20× magnification. The number of cells was quantified using the “Cyto3” and “livecell_cp3” models in Cellpose software [26].

### Xenograft tumor model

Lentiviral vectors encoding short hairpin RNA (shRNA) sequences targeting PPP1R18 (sh-PPP1R18) or a non-targeting control shRNA (sh-NC) were generated by GeneChem Co. (Shanghai, China). The target sequence for sh-PPP1R18 was 5’′-GAGAAGAAAGACAAGACTACT-3′, and the target sequence for sh-NC was 5′-TTCTCCGAACGTGTCACGT-3′. 786-O cells were transduced with lentiviral particles for 24 h and subsequently selected with puromycin (2 µg/mL) for 72 h to establish stable PPP1R18-knockdown and control cell lines.

All animal studies were approved by the Ethics Committee and conducted in accordance with animal welfare guidelines. Male NDG mice (5 weeks old; *n* = 10) were randomly assigned to two groups (*n* = 5 per group). A total of 1 × 10⁷ stably transfected 786-O cells (sh-NC or sh-PPP1R18) suspended in 100 µL of serum-free DMEM supplemented with 50% Matrigel (Corning, Inc.) were subcutaneously injected into the right flank of each mouse. Tumor growth was monitored every three days, and tumor volume was calculated using the formula: V = (length × width²)/2. After three weeks, the mice were euthanized, and tumors were excised, weighed, and photographed. Tumor specimens were subsequently fixed in 4% paraformaldehyde, embedded in paraffin, and sectioned for immunohistochemical (IHC) analysis.

### Immunohistochemistry (IHC)

Immunohistochemistry (IHC) was performed using the PV-6000 Polymer Detection System (ZSGB-BIO, Beijing, China) according to the manufacturer’s instructions. Paraffin-embedded tumor sections were deparaffinized in xylene and rehydrated through a graded ethanol series. Antigen retrieval was performed by boiling the sections in citrate buffer (pH 6.0) for 20 min. Endogenous peroxidase activity was blocked by incubating sections with the endogenous peroxidase blocking reagent provided in the kit for 10 min at room temperature. The sections were then incubated overnight at 4 °C with primary antibodies against PPP1R18 (Santa Cruz Biotechnology, sc-376816, 1:100) and Ki-67 (Proteintech, 27309-1-AP, 1:5000). After washing with PBS (3 × 3 min), sections were incubated with the enzyme-labeled goat anti-mouse/rabbit IgG polymer reagent (PV-6000) for 20 min at room temperature. For visualization, freshly prepared DAB working solution was applied for 3–5 min at room temperature under microscopic monitoring, and the reaction was stopped by rinsing with tap water. Sections were counterstained with hematoxylin for 60 s, differentiated, blued, dehydrated, cleared, and mounted. Images were acquired using an inverted microscope (Olympus).

### Statistical analysis

Statistical analyses were performed using GraphPad Prism (version 10.0, GraphPad Software, La Jolla, CA, USA) and R software (version 4.3.1). Comparisons between two groups were performed using the Student’s t-test or Wilcoxon test, depending on data distribution. For comparisons among multiple groups, One-way ANOVA or the Kruskal-Wallis test was used. Categorical variables were compared using the Chi-square test or Fisher’s exact test. A logistic regression model was employed to evaluate the association between PPP1R18 expression and clinicopathological features. Survival curves were generated using the Kaplan-Meier method and compared with the log-rank test. Univariate and multivariate Cox proportional hazards models were used to identify prognostic factors. Variables with a p-value < 0.1 in the univariate analysis were included in the multivariate model. A two-sided p-value < 0.05 was considered statistically significant.

## Results

### PPP1R18 is upregulated in ccRCC

**Fig. 1:**
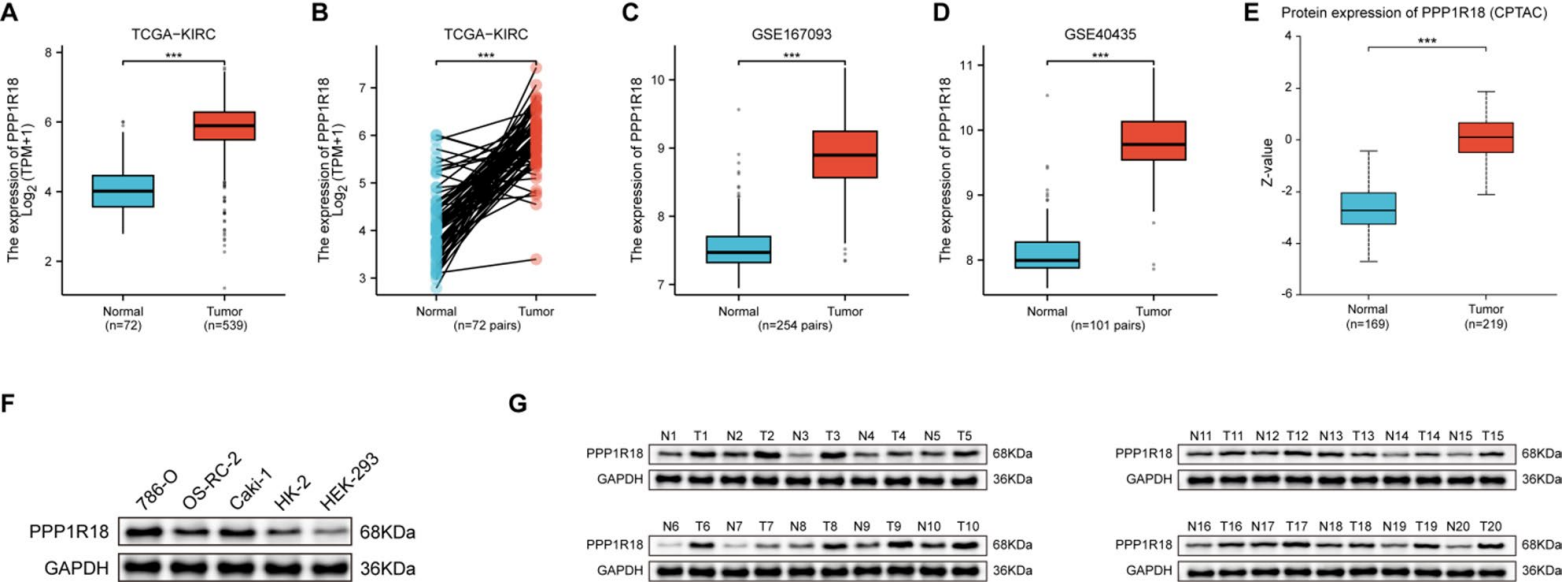
Expression levels of PPP1R18 in ccRCC. (**A**) Differential mRNA expression of PPP1R18 in ccRCC (*n* = 539) and normal kidney tissues (*n* = 72) from the TCGA database. (**B-D**) Paired analysis of PPP1R18 mRNA levels in ccRCC and adjacent normal tissues from TCGA (*n* = 72 pairs) (**B**), GSE167093 (*n* = 254 pairs) (**C**), and GSE40435 (*n* = 101 pairs) (**D**) datasets. (**E**) Protein expression of PPP1R18 in ccRCC and normal kidney tissues from the CPTAC database. (**F**) Protein levels of PPP1R18 in ccRCC cell lines and normal kidney cell lines. (**G**) Representative Western blot analysis of PPP1R18 protein levels in 20 pairs of ccRCC tumors (T) and their adjacent normal tissues (N). ***p < 0.001

To assess the clinical relevance of PPP1R18, we first analyzed its expression in multiple public databases. In the TCGA-KIRC dataset, PPP1R18 mRNA levels were significantly higher in ccRCC tumors compared to normal kidney tissues (*n* = 539 vs. *n* = 72; *P* < 0.001) and in paired tumor tissues compared to adjacent non-tumorous tissues (*n* = 72 pairs; *P* < 0.001) (Fig. 1A, B). Similarly, analyses of two independent GEO datasets, GSE167093 (*n* = 254 pairs) and GSE40435 (*n* = 101 pairs), consistently showed significantly elevated PPP1R18 mRNA expression in ccRCC tissues (Fig. 1C, D). At the protein level, data from the CPTAC database also revealed a significant upregulation of PPP1R18 in ccRCC tumors relative to normal tissues (Fig. 1E).

We next validated these findings at the protein level using Western blotting. PPP1R18 protein expression was substantially higher in a panel of ccRCC cell lines (786-O, OSRC-2, and Caki-1) than in normal renal cell lines (HK-2, HEK-293) (Fig. 1F). Furthermore, in a cohort of 20 paired clinical specimens, PPP1R18 protein levels were markedly increased in ccRCC tissues compared to their matched adjacent non-tumorous tissues (Fig. 1G). Collectively, these data demonstrate that PPP1R18 is overexpressed in ccRCC.

### High PPP1R18 expression correlates with aggressive clinicopathological features and poor prognosis

**Fig. 2:**
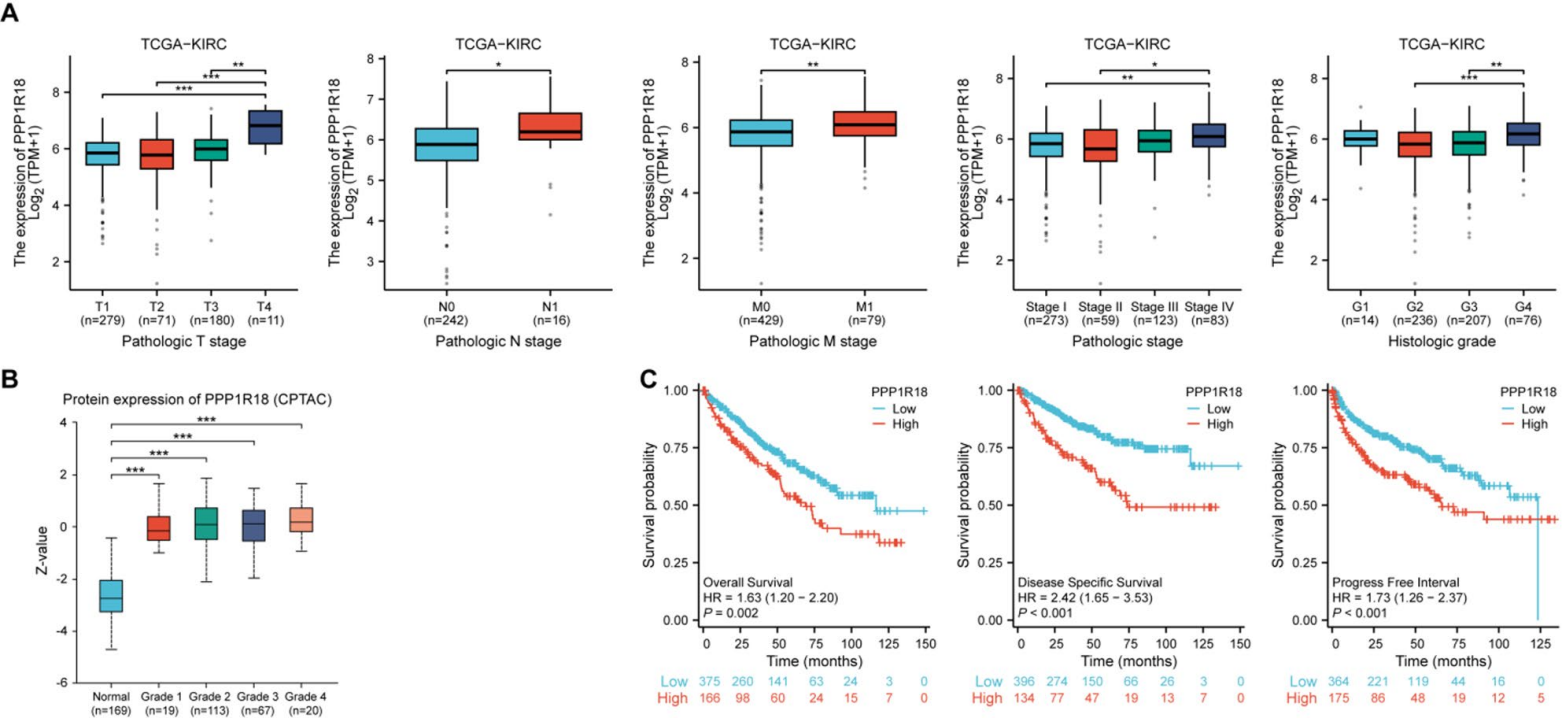
Clinical and prognostic significance of PPP1R18 expression in ccRCC. (**A**) Correlation of PPP1R18 mRNA expression with T stage, N stage, M stage, histological grade, and pathological stage in the TCGA cohort. (**B**) Correlation between PPP1R18 protein expression and histological grade in the CPTAC cohort. (**C**) Kaplan-Meier analysis of Overall Survival (OS), Disease-Specific Survival (DSS), and Progression-Free Interval (PFI) based on PPP1R18 mRNA expression levels. *p < 0.05, **p < 0.01, ***p < 0.001

We next investigated the association between PPP1R18 expression and the clinical features of ccRCC patients using data from the TCGA-KIRC and CPTAC cohorts. In the TCGA cohort, high PPP1R18 expression was significantly associated with advanced T stage, N stage, M stage, pathological stage, and higher histological grade (Fig. 2A). A similar trend was observed in the CPTAC cohort, where high PPP1R18 expression was associated with a higher histological grade, although this did not reach statistical significance (Fig. 2B).

Logistic regression analysis further revealed that high PPP1R18 expression was a significant predictor for advanced T stage (OR = 1.54, 95% CI: 1.08–2.20), distant metastasis (OR = 1.90, 95% CI: 1.16–3.12), advanced pathological stage (OR = 1.56, 95% CI: 1.10–2.21), and higher histological grade (OR = 2.26, 95% CI: 1.35–3.77) (Table 1). Kaplan-Meier analysis demonstrated that patients with high PPP1R18 expression had significantly shorter overall survival (OS; HR = 1.63, 95% CI: 1.20–2.20), disease-specific survival (DSS; HR = 2.42, 95% CI: 1.65–3.53), and progression-free interval (PFI; HR = 1.73, 95% CI: 1.26–2.37) (Fig. 2C). Moreover, in univariate Cox regression analysis, high PPP1R18 expression emerged as an independent prognostic factor for OS (Table 2).

**Table 1:** Logistic analysis of PPP1R18 and clinical characteristics in ccRCC

Characteristics	Total (*N*)	OR (95% CI)	*P* value
T stage (T3&T4 vs. T1&2)	541	1.543 (1.081–2.201)	**0.017**
N stage (N1 vs. N0)	258	3.153 (0.989–10.050)	0.052
M stage (M1 vs. M0)	508	1.902 (1.159–3.120)	**0.011**
Stage (Stage III&IV vs. Stage I&II)	538	1.557 (1.097–2.210)	**0.013**
Grade (G4 vs. G1&2&3)	533	2.256 (1.351–3.767)	**0.002**

Bold values indicate statistical significance (*P﻿* < 0.05)

**Table 2:** Cox regression analysis of variables for OS in ccRCC

Characteristics	Total(*N*)	Univariate analysis			Multivariate analysis	
		Hazard ratio (95% CI)	*P* value		Hazard ratio (95% CI)	*P* value
Age	541					
<= 60	269	Reference			Reference	
> 60	272	1.791 (1.319–2.432)	**< 0.001**		1.778 (1.159–2.727)	**0.008**
Gender	541					
Female	187	Reference				
Male	354	0.924 (0.679–1.257)	0.613			
T stage	541					
T1&T2	350	Reference			Reference	
T3&T4	191	3.210 (2.373–4.342)	**< 0.001**		1.733 (1.080–2.782)	**0.023**
N stage	258					
N0	242	Reference			Reference	
N1	16	3.422 (1.817–6.446)	**< 0.001**		1.670 (0.835–3.341)	0.147
M stage	508					
M0	429	Reference			Reference	
M1	79	4.401 (3.226–6.002)	**< 0.001**		2.828 (1.753–4.564)	**< 0.00**1
Grade	533					
G1&G2	250	Reference			Reference	
G3&G4	283	2.665 (1.898–3.743)	**< 0.001**		1.643 (1.004–2.688)	**0.048**
PPP1R18	541	1.444 (1.155–1.806)	**0.001**		1.315 (0.931–1.856)	0.120

Bold values indicate statistical significance (*P* < 0.05)

### PPP1R18 is essential for ccRCC cell proliferation, migration, and invasion in vitro

**Fig. 3:**
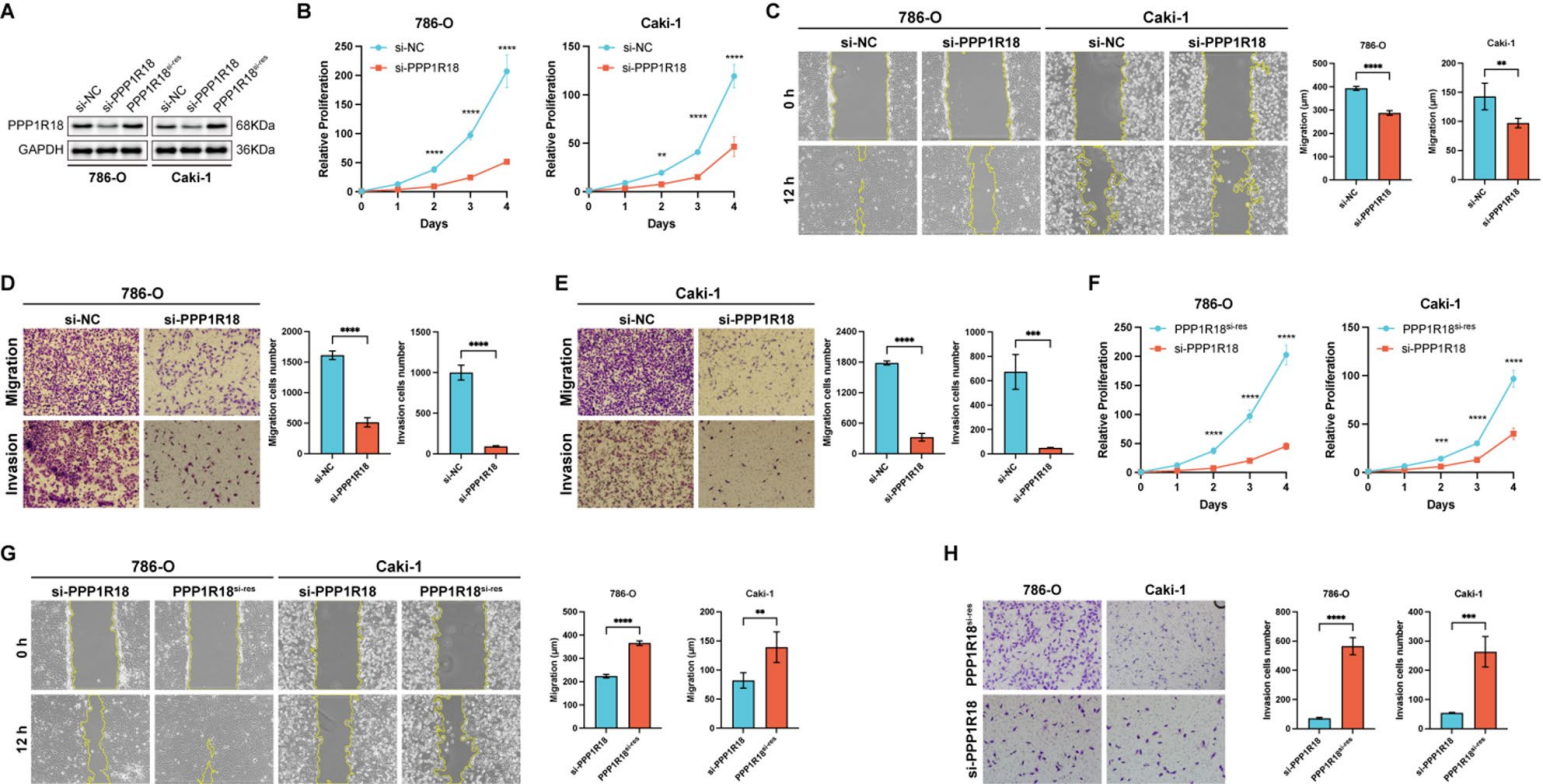
Functional assays following PPP1R18 knockdown and re-expression in ccRCC cells. (**A**) Western blot analysis assessing the knockdown and re-expression efficiency of PPP1R18 in 786-O and Caki-1 cells. (**B**) CCK-8 assay of cell viability in 786-O and Caki-1 cells transfected with si-NC or si-PPP1R18. (**C**) Wound healing assay in 786-O and Caki-1 cells after PPP1R18 knockdown. (**D-E**) Transwell migration and invasion assays in 786-O (**D**) and Caki-1 (**E**) cells after PPP1R18 knockdown. (**F-H**) Rescue experiments using an siRNA-resistant PPP1R18 construct (PPP1R18^si−res^) in PPP1R18-knockdown cells, including proliferation (**F**), migration (**G**), and invasion (**H**) assays. All quantitative data are presented as the mean ± SD from at least three independent experiments. Images are representative of at least three independent experiments. **p < 0.01, ***p < 0.001, ****p < 0.0001

To explore the biological functions of PPP1R18 in ccRCC, we performed loss-of-function experiments in 786-O and Caki-1 cells, which exhibit high endogenous PPP1R18 expression. Transfection with PPP1R18-specific siRNA (si-PPP1R18) effectively reduced its protein expression compared to the negative control (si-NC) (Fig. 3A). The CCK-8 assay showed that PPP1R18 knockdown significantly impaired the proliferative capacity of both 786-O and Caki-1 cells (Fig. 3B). Furthermore, the wound healing assay revealed that the migratory ability of cells was substantially reduced following PPP1R18 depletion (Fig. 3C). Consistent with these findings, Transwell assays demonstrated that PPP1R18 knockdown significantly inhibited both the migration and invasion of 786-O and Caki-1 cells (Fig. 3D, E). These results suggest that PPP1R18 is crucial for maintaining the malignant phenotypes of ccRCC cells.

To further validate that these malignant phenotypes were directly mediated by PPP1R18, we conducted functional rescue assays by reintroducing an siRNA-resistant PPP1R18 construct (PPP1R18^si−res^) into cells with endogenous PPP1R18 silenced. As shown in Fig. 3F-H, re-expression of PPP1R18 markedly restored cell proliferation, migration, and invasion that had been suppressed by PPP1R18 knockdown. These findings provide robust evidence that PPP1R18 acts as a direct and essential driver of aggressive phenotypes in ccRCC cells.

### PPP1R18 promotes EMT and activates the Wnt/β-catenin signaling pathway

**Fig. 4:**
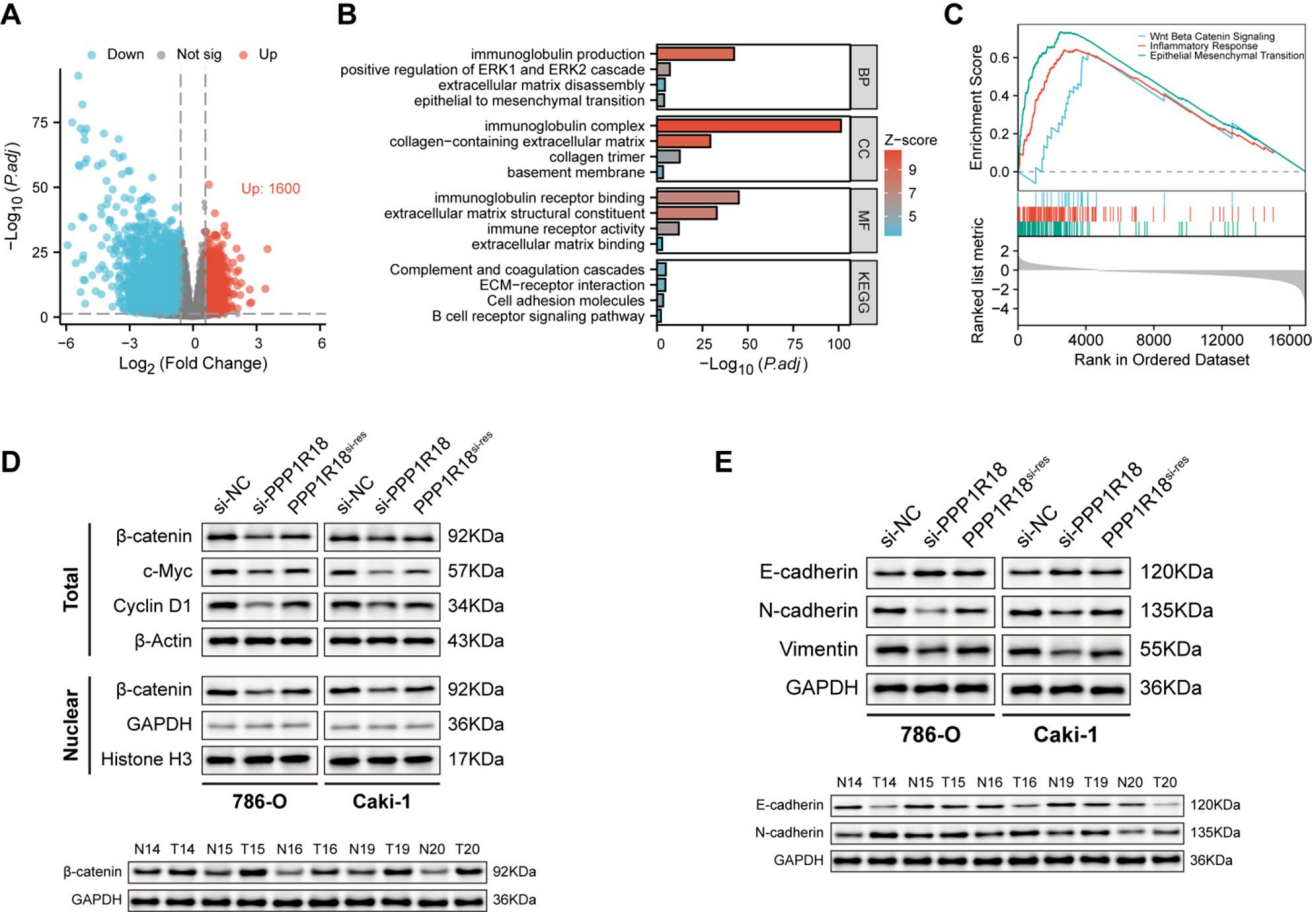
Enrichment analysis and effect of PPP1R18 on Wnt/β-catenin and EMT signaling. (**A**) Volcano plot showing differentially expressed genes (DEGs) between the high and low PPP1R18 expression groups in the TCGA-KIRC dataset (logFC > 0.585 and p.adj < 0.05). (**B**) Gene Ontology (GO) and Kyoto Encyclopedia of Genes and Genomes (KEGG) enrichment analysis of the DEGs. (**C**) Gene Set Enrichment Analysis (GSEA) of the DEGs. (**D**) Western blot analysis showing decreased levels of Wnt/β-catenin pathway-related proteins after PPP1R18 knockdown in 786-O and Caki-1 cells, and the rescue of their expression by an siRNA-resistant PPP1R18 construct (PPP1R18^si−res^). The lower blots show β-catenin expression in five paired samples of ccRCC tumors (T) and adjacent normal tissues (N). (**E**) Western blot analysis of Wnt/β-catenin pathway-related proteins in 786-O and Caki-1 cells following PPP1R18 knockdown and rescue with PPP1R18^si−res^. The lower blots show representative expression of E-cadherin and N-cadherin in five paired samples of ccRCC tumors (T) and adjacent normal tissues (N). The Western blot results are representative of three independent experiments

To investigate the molecular mechanisms underlying the oncogenic role of PPP1R18, we performed enrichment analyses. A total of 1600 DEGs were identified (Fig. 4A). KEGG and GO analyses indicated that these DEGs were highly enriched in pathways related to immune response and epithelial-mesenchymal transition (EMT) (Fig. 4B). GSEA further confirmed significant enrichment in “Wnt Beta Catenin Signaling” and “Epithelial Mesenchymal Transition” hallmark gene sets (Fig. 4C).

Based on these bioinformatic findings, we hypothesized that PPP1R18 promotes ccRCC progression by activating the Wnt/β-catenin pathway and inducing EMT. Western blot analysis showed that knockdown of PPP1R18 in 786-O and Caki-1 cells led to a marked decrease in the protein levels of both total and nuclear β-catenin, as well as its downstream targets, c-Myc and Cyclin D1 (Fig. 4D). Concurrently, PPP1R18 depletion resulted in an upregulation of the epithelial marker E-cadherin and a downregulation of the mesenchymal markers N-cadherin and vimentin (Fig. 4E). These results indicate that PPP1R18 knockdown inhibits Wnt/β-catenin signaling and reverses the EMT process in ccRCC cells.

To further verify that the inhibition of Wnt/β-catenin signaling and the reversal of EMT were specifically attributable to PPP1R18 depletion, we performed a rescue experiment by reintroducing an siRNA-resistant PPP1R18 construct (PPP1R18^si−res^) into cells in which endogenous PPP1R18 had been silenced. As shown in Fig. 4D, exogenous expression of PPP1R18^si−res^ effectively restored the protein levels of total and nuclear β-catenin, along with its downstream effectors c-Myc and Cyclin D1. Consistently, re-expression of PPP1R18^si−res^ also reversed the alterations in EMT markers, resulting in reduced E-cadherin expression and increased levels of N-cadherin and vimentin (Fig. 4E). Together, these findings provide compelling evidence that PPP1R18 directly modulates the Wnt/β-catenin pathway and EMT program in ccRCC cells.

### PPP1R18 regulates Wnt/β-catenin signaling and EMT via the ERK pathway

**Fig. 5:**
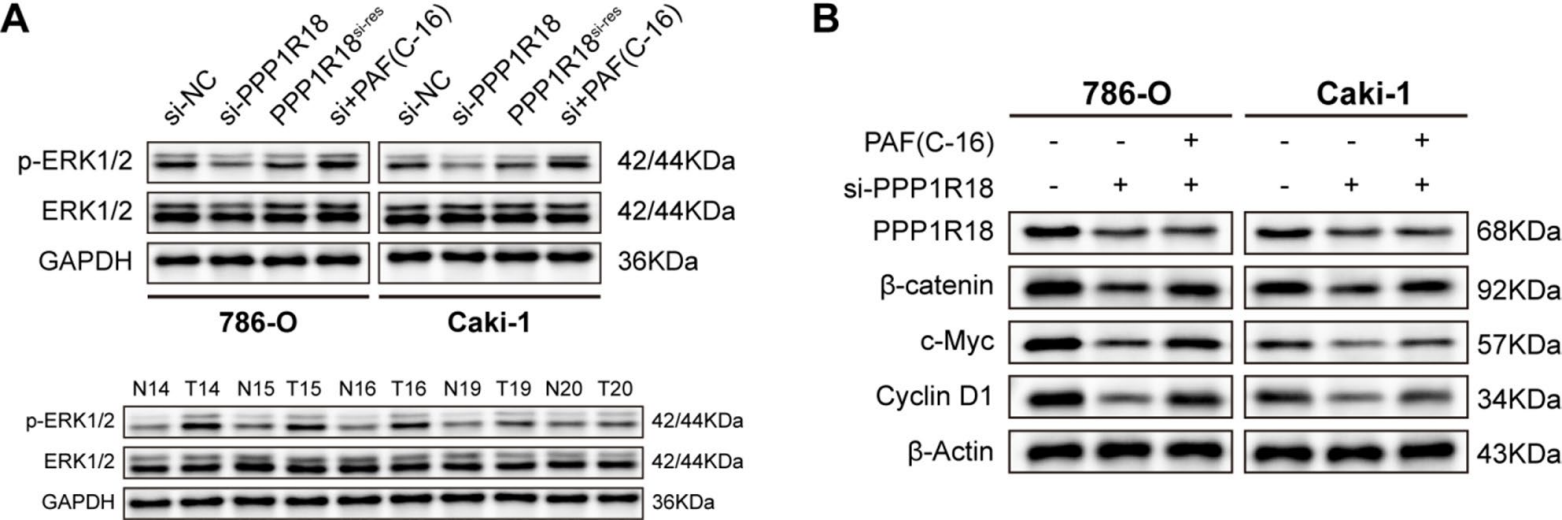
Regulation of Wnt/β-catenin signaling via the ERK pathway. (**A**) Western blot analysis of phospho-ERK (p-ERK) and total ERK in 786-O and Caki-1 cells under the indicated conditions (PPP1R18 knockdown, PPP1R18^si−res^ rescue, and ERK agonist PAF(C-16) treatment). The lower blots show representative expression of p-ERK and total ERK in 5 pairs of ccRCC tumors (T) and adjacent normal tissues (N). (**B**) Western blot analysis of Wnt/β-catenin pathway proteins in cells treated with si-PPP1R18 and PAF(C-16). The results are representative of three independent experiments

Our GO analysis also revealed an enrichment in the “positive regulation of ERK1 and ERK2 cascade” (Fig. 4B), and a previous study implicated the ERK pathway in PPP1R18-mediated effects [21]. We therefore examined whether ERK signaling was involved in PPP1R18’s function in ccRCC. We found that PPP1R18 knockdown significantly suppressed the phosphorylation of ERK (p-ERK) in both 786-O and Caki-1 cells, while total ERK levels remained unchanged. To confirm the specific action of the ERK agonist used for rescue experiments, we treated PPP1R18-depleted cells with PAF(C-16) and observed a restoration of p-ERK levels without any significant change in total ERK (Fig. 5A).

**Fig. 6:**
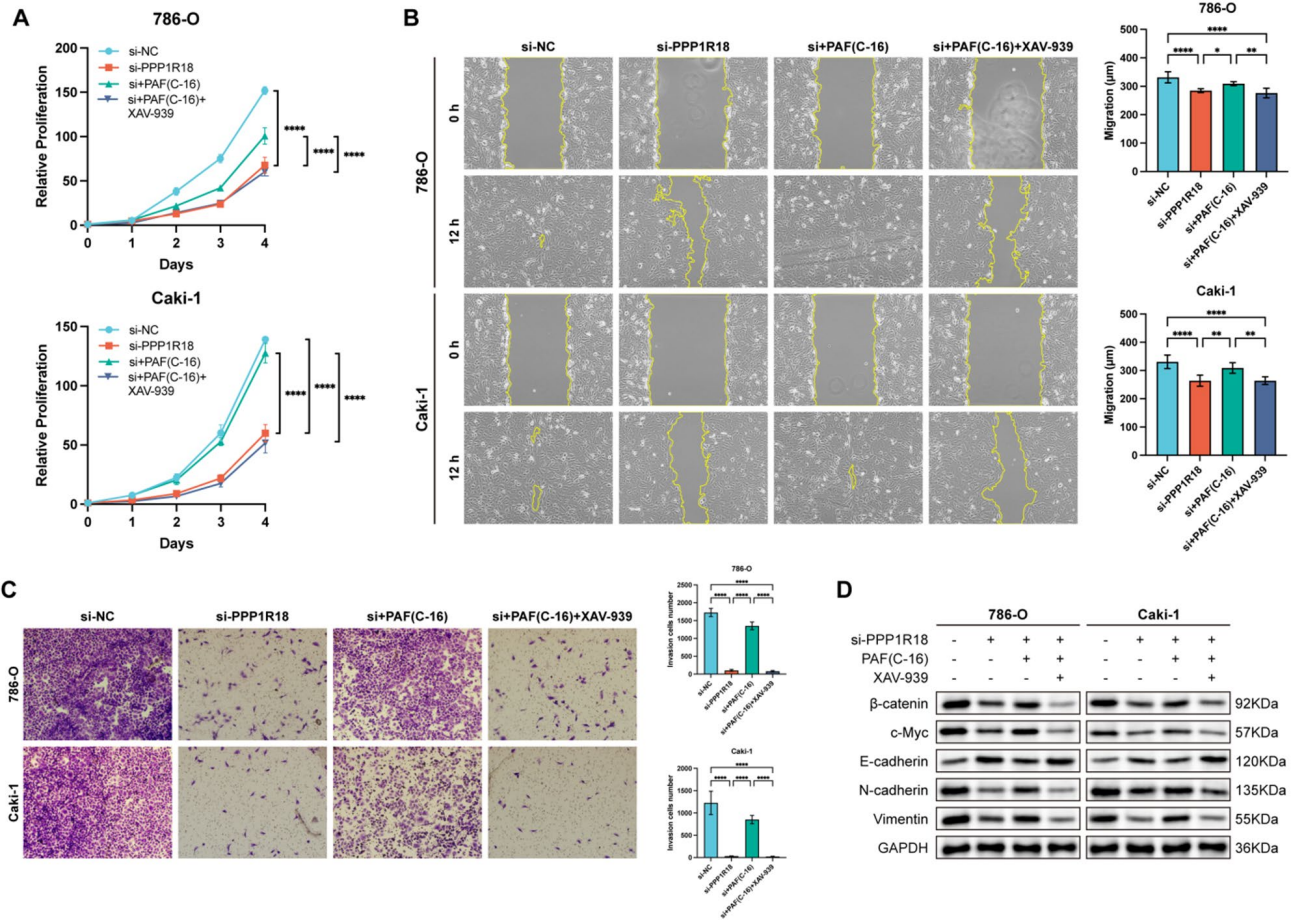
Role of the ERK/Wnt/β-catenin axis in PPP1R18-mediated progression. (**A-C**) Analysis of cell proliferation (**A**), migration (**B**), and invasion (**C**) in PPP1R18-knockdown cells treated with the ERK agonist PAF(C-16) and/or the Wnt/β-catenin inhibitor XAV-939. (**D**) Western blot analysis of Wnt/β-catenin pathway proteins and EMT markers in PPP1R18-knockdown cells treated with PAF(C-16) with or without XAV-939. All quantitative data are presented as the mean ± SD from at least three independent experiments. Images are representative of at least three independent experiments. *p < 0.05, **p < 0.01, ***p < 0.001, ****p < 0.0001

To determine if ERK activation is downstream of PPP1R18 and mediates its effects on Wnt/β-catenin and EMT, we performed a rescue experiment. Treatment with PAF(C-16), an ERK pathway agonist, restored the expression of total β-catenin, c-Myc and Cyclin D1 in PPP1R18-depleted cells (Fig. 5B). Importantly, we confirmed that treatment with the ERK agonist did not affect the suppressed protein levels of PPP1R18 itself, providing direct evidence that PPP1R18 functions upstream of ERK signaling (Fig. 5B). Similarly, PAF(C-16) treatment reversed the effects of PPP1R18 knockdown on EMT markers, causing a decrease in E-cadherin and an increase in N-cadherin and vimentin expression (Fig. 6D). These findings suggest that PPP1R18 regulates the Wnt/β-catenin pathway and EMT through the activation of ERK signaling.

### Activation of the ERK/Wnt/β-catenin axis is essential for PPP1R18-mediated ccRCC progression

Finally, we conducted further rescue experiments to solidify the hierarchical relationship within the PPP1R18/ERK/Wnt/β-catenin axis. As expected, treatment with the ERK agonist PAF(C-16) rescued the proliferation, migration, and invasion abilities of PPP1R18-knockdown cells (Fig. 6A-C). To test if this rescue effect was dependent on Wnt/β-catenin signaling, we co-treated the cells with XAV-939, a Wnt/β-catenin inhibitor. The addition of XAV-939 abrogated the pro-proliferative, -migratory, and -invasive effects induced by PAF(C-16) in PPP1R18-depleted cells (Fig. 6A-C). Western blot analysis confirmed that XAV-939 co-treatment blocked the PAF(C-16)-induced restoration of Wnt/β-catenin and EMT marker expression (Fig. 6D). Taken together, these results demonstrate that PPP1R18 promotes the malignant phenotypes of ccRCC cells by activating the Wnt/β-catenin pathway in an ERK-dependent manner.

### Knockdown of PPP1R18 inhibits ccRCC tumor growth in vivo

**Fig. 7:**
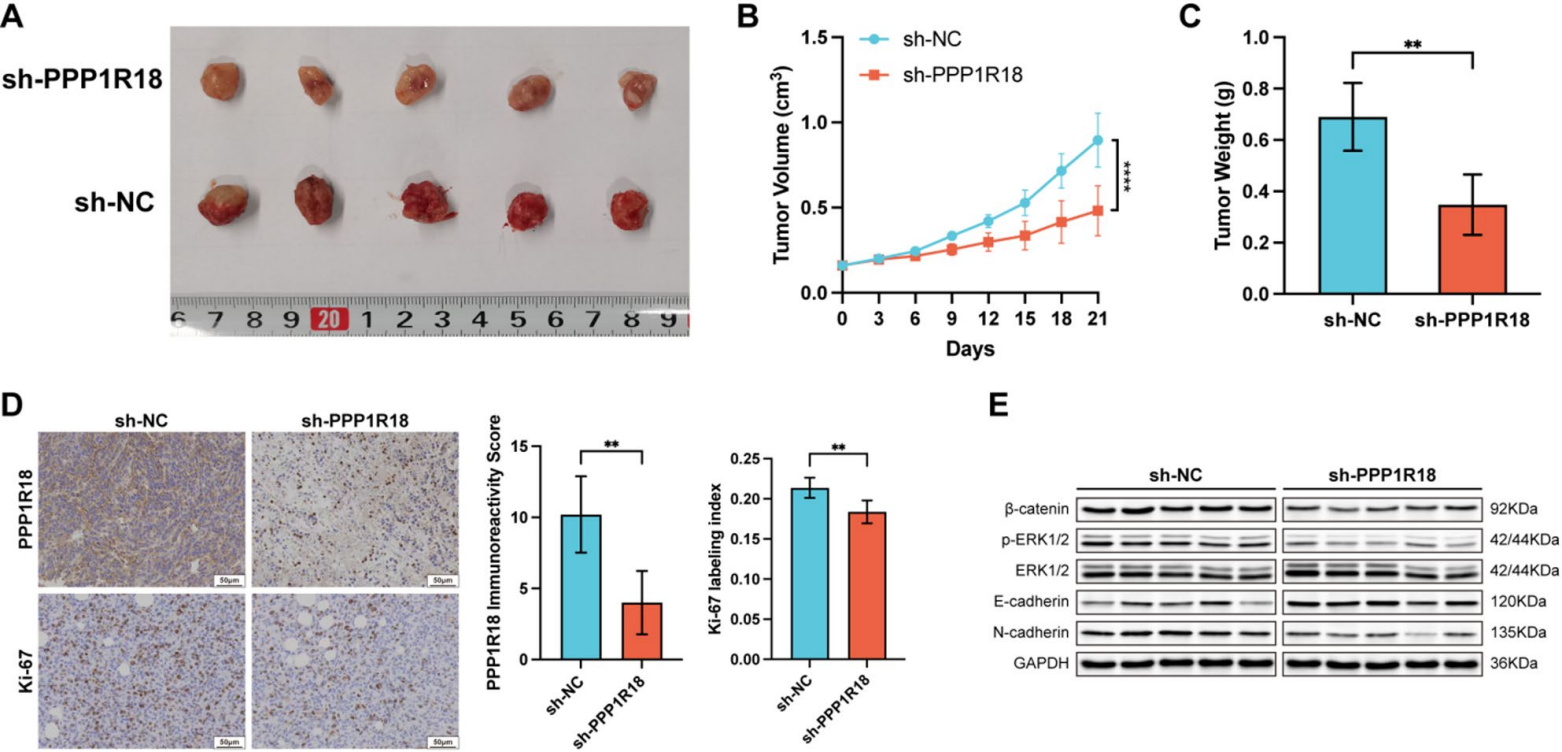
Effect of PPP1R18 knockdown on ccRCC tumor growth in vivo. (**A**) Representative images of tumors from the sh-NC and sh-PPP1R18 groups (*n* = 5 per group). (**B**) Tumor growth curves for the sh-NC and sh-PPP1R18 groups. Tumor volumes were measured every 3 days. (**C**) Final tumor weights at the end of the experiment. (**D**) Representative immunohistochemistry (IHC) staining images for PPP1R18 and Ki-67 in xenograft tumor tissues from both groups. (**E**) Western blot analysis of β-catenin, p-ERK, N-cadherin, and E-cadherin protein levels in xenograft tumor tissues. Data are presented as the mean ± SD (*n* = 5 mice per group). Western blot images are representative of tumor samples from the respective groups. **p < 0.01, ****p < 0.0001

To further validate the oncogenic role of PPP1R18 in vivo, we established a subcutaneous xenograft model in NDG mice using 786-O cells stably expressing sh-PPP1R18 or sh-NC. As shown in Fig. 7A-C, the tumors derived from PPP1R18-knockdown cells grew significantly slower compared to the control group. At the end of the experiment (day 21), the average tumor volume and weight in the sh-PPP1R18 group were substantially lower than those in the sh-NC group. Furthermore, IHC staining of the excised tumor tissues confirmed lower PPP1R18 expression in the knockdown group and revealed a significant reduction in the percentage of Ki-67-positive cells, indicating decreased cell proliferation in vivo (Fig. 7D). To further validate the molecular mechanism in vivo, we extracted proteins from the xenograft tumor tissues for Western blot analysis. Consistent with our in vitro observations, tumors in the sh-PPP1R18 group exhibited significantly reduced levels of p-ERK, total $\beta$-catenin, and the mesenchymal marker N-cadherin, alongside a marked upregulation of the epithelial marker E-cadherin compared to the sh-NC group (Fig. 7E). These results collectively demonstrate that PPP1R18 is crucial for ccRCC tumor growth in vivo.

### The PPP1R18/ERK/Wnt/β-catenin axis is validated in ccRCC clinical specimens

To confirm that the signaling pathway identified in vitro is clinically relevant, we performed Western blot analysis on 5 pairs of ccRCC and adjacent normal tissues selected for their high PPP1R18 expression. Consistent with our cellular experiments, tumor tissues showed a marked increase in the phosphorylation of ERK compared to their matched normal counterparts (Fig. 5A). Furthermore, these PPP1R18-overexpressing tumors also exhibited significantly elevated levels of total β-catenin (Fig. 4D). In line with an induced EMT phenotype, the tumor samples displayed decreased expression of the epithelial marker E-cadherin and increased expression of the mesenchymal marker N-cadherin (Fig. 4E). Collectively, these clinical data provide strong evidence supporting the existence of the PPP1R18/ERK/Wnt/β-catenin/EMT signaling axis in ccRCC patients, corroborating our in vitro findings.

## Discussion

Clear cell renal cell carcinoma (ccRCC) is the most prevalent renal malignancy in adults, with a steadily increasing incidence [1–6]. Despite advances in targeted therapy and immunotherapy, the prognosis for patients with advanced ccRCC remains poor [17, 27]. Therefore, elucidating the key molecular drivers of ccRCC progression and their regulatory networks is crucial for developing novel diagnostic markers and therapeutic targets to improve clinical management [28, 29]. While previous bioinformatic studies have correlated high PPP1R18 expression with poor prognosis, our study provided comprehensive functional validation establishing PPP1R18 as a critical oncogenic driver in ccRCC [23]. We systematically demonstrated that PPP1R18 is significantly upregulated in ccRCC tissues and cell lines, and its high expression is strongly associated with adverse clinicopathological features and poor outcomes, serving as an independent prognostic factor. Functionally, we found that PPP1R18 promotes ccRCC progression by enhancing cell proliferation, migration, and invasion. Mechanistically, we have uncovered a novel signaling pathway whereby PPP1R18 activates the ERK pathway, which subsequently triggers Wnt/β-catenin signaling and induces epithelial-mesenchymal transition (EMT), ultimately fostering ccRCC malignancy. These findings offer new insights into the molecular pathogenesis of ccRCC and present PPP1R18 as a potential therapeutic target.

Our investigation began with a comprehensive analysis of multiple public databases, which consistently revealed that both mRNA and protein levels of PPP1R18 were significantly elevated in ccRCC tissues compared to normal renal tissues. This observation was further validated in our institutional cohort of clinical specimens and in ccRCC cell lines. The consistency across these independent cohorts suggests that PPP1R18 overexpression is a robust molecular feature of ccRCC. We then found that high PPP1R18 expression was significantly associated with aggressive clinicopathological characteristics, including advanced tumor stage, higher grade, and distant metastasis. Critically, our survival analyses identified high PPP1R18 expression as an independent predictor of shorter overall survival, disease-specific survival, and progression-free interval. These results align with several recent bioinformatics-based studies and suggest that PPP1R18 likely functions as an oncogene associated with poor prognosis in ccRCC [22, 23].

By performing loss-of-function studies in ccRCC cell lines, we provided the first functional evidence for the role of PPP1R18. We demonstrated that inhibiting PPP1R18 expression significantly impaired the proliferative, migratory, and invasive capacities of ccRCC cells. Crucially, these in vitro findings were further substantiated by an in vivo xenograft model, where stable knockdown of PPP1R18 markedly suppressed tumor growth. These functional data, combined with our expression and prognostic analyses, provide compelling evidence that PPP1R18 is a key driver actively involved in promoting malignant phenotypes. Collectively, our study characterizes PPP1R18 not merely as a potential biomarker, but as a functionally validated oncogenic driver in ccRCC.

To uncover the molecular mechanisms underlying the oncogenic effects of PPP1R18, we conducted bioinformatic analyses of the TCGA-KIRC dataset. Our results pointed to a significant enrichment of genes associated with the Wnt/β-catenin signaling pathway and EMT. The Wnt/β-catenin pathway is a well-established oncogenic driver in numerous cancers, including ccRCC, where its aberrant activation is linked to tumor initiation and progression [30–32]. EMT, a critical biological process by which tumor cells acquire motility and invasiveness, is a hallmark of metastasis and is frequently regulated by Wnt/β-catenin signaling [33–35]. Our experimental data substantiated these bioinformatic predictions. Knockdown of PPP1R18 led to a marked reduction in key components of the Wnt/β-catenin pathway, including β-catenin, c-Myc, and Cyclin D1. Concurrently, we observed a reversal of the EMT phenotype, characterized by the upregulation of the epithelial marker E-cadherin and downregulation of the mesenchymal markers N-cadherin and vimentin. These findings strongly indicate that PPP1R18 promotes ccRCC progression, at least in part, by activating the Wnt/β-catenin pathway and inducing EMT.

A core mechanistic finding of our study is the identification of a novel signaling cascade in ccRCC. While a link between PPP1R18 and ERK has been reported in esophageal cancer, our study is the first to establish this connection in ccRCC and, more importantly, to uncover the novel downstream signaling axis whereby PPP1R18-mediated ERK activation triggers the Wnt/β-catenin pathway to induce EMT [21]. This was initially suggested by our GO enrichment analysis and subsequently confirmed by our finding that PPP1R18 knockdown significantly inhibited ERK phosphorylation. This is a crucial discovery, as constitutive activation of the ERK pathway is itself an independent factor associated with poor prognosis in RCC [36, 37]. While our study clearly demonstrates this regulatory effect, the precise molecular link between PPP1R18 and ERK requires further investigation. Based on existing literature, we hypothesize that PPP1R18 may function as a scaffold protein to facilitate Src-mediated activation of the ERK pathway, given that PPP1R18 is a known binding partner of Src, a classic upstream activator of the Ras/Raf/MEK/ERK cascade [18, 21, 38–40]. More importantly, through a series of loss-of-function and rescue experiments, we delineated a clear signaling cascade from PPP1R18 to ERK, then to Wnt/β-catenin, culminating in malignant phenotypes. We showed that activating the ERK pathway with an agonist, PAF(C-16), rescued the inhibition of Wnt/β-catenin signaling caused by PPP1R18 knockdown, placing Wnt/β-catenin downstream of ERK in this context. The subsequent abrogation of this rescue effect by a Wnt/β-catenin inhibitor, XAV-939, further solidified that PPP1R18 exerts its oncogenic functions primarily through the sequential activation of the ERK and Wnt/β-catenin pathways.

The crosstalk between the ERK and Wnt/β-catenin pathways, while reported in other cancers, has not been previously described in ccRCC, particularly under the regulation of PPP1R18 [41–45]. A well-established mechanism for this crosstalk involves the direct regulation of Glycogen Synthase Kinase 3β (GSK3β), the master kinase of the β-catenin destruction complex [46–51]. In the canonical Wnt-off state, GSK3β, in a complex with Axin and APC, phosphorylates β-catenin, marking it for ubiquitination and proteasomal degradation. Our data strongly suggest that PPP1R18-mediated ERK activation interrupts this process. Activated ERK (p-ERK) is known to directly phosphorylate GSK3β at an inhibitory serine residue (Ser9), leading to its inactivation. The inactivation of GSK3β disrupts the integrity and function of the destruction complex, thereby preventing β-catenin phosphorylation. As a result, unphosphorylated, stable β-catenin accumulates in the cytoplasm, translocates to the nucleus, and activates the transcription of its target genes, including c-Myc and Cyclin D1 [35, 46, 47, 52–55]. This provides a compelling and detailed molecular explanation for our observations that PPP1R18 knockdown, by suppressing ERK phosphorylation, led to a significant decrease in both total and nuclear β-catenin, and a corresponding reduction in its downstream targets. As c-Myc and Cyclin D1 are key drivers of cell proliferation, this directly links the PPP1R18/ERK/Wnt/β-catenin axis to the proliferative phenotype of ccRCC [56–58]. Likewise, the activation of the Wnt/β-catenin/EMT axis accounts for the invasive characteristics we observed, linking this signaling cascade to ccRCC metastasis [34, 35].

A notable observation from our bioinformatic analysis was the pronounced enrichment of immune-related GO/KEGG terms among differentially expressed genes. Such findings are frequently reported in bulk tumor transcriptome studies and likely reflect the intricate interplay between malignant cells and the tumor microenvironment (TME) [59–61]. While the primary focus of this work is the cell-intrinsic PPP1R18/ERK/Wnt/β-catenin axis, potential crosstalk with the immune compartment warrants investigation. Future studies aimed at elucidating how PPP1R18-driven signaling in cancer cells influences immune infiltration and function and may reveal therapeutic opportunities.

Our study demonstrates the functional importance of the PPP1R18/ERK/Wnt/β-catenin signaling axis in regulating the malignant phenotypes of ccRCC. This newly identified axis enhances our understanding of the molecular pathogenesis of ccRCC and may offer new avenues for therapeutic intervention. Although PPP1R18 is a regulatory subunit rather than an enzyme, several therapeutic avenues can be envisioned. First, direct inhibition may be achieved through small-molecule inhibitors or peptidomimetics designed to disrupt its interaction with key partners, such as the PP1 catalytic subunit or components of the ERK activation complex, an approach that has become increasingly feasible with recent advances in targeting protein-protein interactions [62]. Second, and perhaps more immediately feasible, is an indirect therapeutic strategy. Given our finding that PPP1R18’s oncogenic effects are mediated through the ERK/Wnt/β-catenin axis, existing inhibitors targeting this downstream pathway could be leveraged. For instance, MEK inhibitors, which are clinically approved for other malignancies, could be effective in ccRCC tumors characterized by high PPP1R18 expression [63]. In this context, PPP1R18 could serve as a predictive biomarker to stratify patients for treatment with downstream pathway inhibitors. Finally, emerging therapeutic modalities such as antisense oligonucleotides or siRNA could be developed to directly target and degrade PPP1R18 mRNA, thereby reducing its protein levels [64]. While direct inhibition of PPP1R18 warrants further investigation, its utility as a biomarker to guide existing therapies represents a promising and actionable clinical strategy.

However, our study has several limitations. First, while we have validated the oncogenic role of PPP1R18 in vivo using a xenograft model, the tumor microenvironment is complex, and further studies are needed to fully understand the role of PPP1R18 in this context [60]. In addition to xenografts, syngeneic or pharmacological inhibition models targeting the Wnt/β-catenin axis in vivo would be valuable to further define the therapeutic potential of PPP1R18 blockade, and represent important directions for future research. Second, the precise molecular mechanism by which PPP1R18 activates the ERK pathway remains to be fully elucidated. Third, although PPP1R18 shows promise as a prognostic biomarker, its clinical utility requires validation in large-scale, prospective, multi-center cohort studies.

## Conclusion

In conclusion, our study identifies PPP1R18 as a critical oncogenic driver and prognostic biomarker in clear cell renal cell carcinoma. We have uncovered a novel signaling axis where PPP1R18 promotes cell proliferation, invasion, and EMT by activating the Wnt/β-catenin pathway in an ERK-dependent manner. This work not only reveals a new dimension of ccRCC pathogenesis but also establishes the PPP1R18-ERK-Wnt/β-catenin axis as a promising therapeutic target for future clinical intervention.

## Supplementary Information

Below is the link to the electronic supplementary material.

Supplementary Material 1

## Data Availability

The data that support the findings of this study are available on request from the corresponding author.

## Abbreviations

ccRCC: Clear cell renal cell carcinoma
RCC: Renal cell carcinoma
EMT: Epithelial-mesenchymal transition
ICIs: Immune checkpoint inhibitors
PPP1R18: Protein phosphatase 1 regulatory subunit 18
PP1: Protein phosphatase 1
TCGA: The cancer genome atlas
GEO: Gene expression omnibus
CPTAC: The clinical proteomic tumor analysis consortium
DEGs: Differentially expressed genes
p.adj: adjusted p-value
GO: Gene Ontology
KEGG: Kyoto encyclopedia of genes and genomes
GSEA: Gene set enrichment analysis
FDR: False discovery rate
WHO: World health organization
ATCC: American type culture collection
FBS: fetal bovine serum
siRNA: Small‑interfering RNA
si-NC: Non-targeting negative control siRNA
shRNA: Short hairpin RNA
SDS-PAGE: Sodium dodecyl-sulfate polyacrylamide gel electrophoresis
PVDF: Polyvinylidene fluoride
CCK-8: Cell counting kit-8
PBS: Phosphate-buffered saline
IHC: Immunohistochemistry
OR: Odds ratio
OS: Overall survival
DSS: Disease-specific survival
PFI: Progression-free interval
HR: Hazard ratio
CI: Confidence interval
GSK3β: Glycogen synthase kinase 3β
