# Supplementary material for: PPP1R18-mediated activation of Wnt/β-catenin and EMT is dependent on ERK signaling in clear cell renal cell carcinoma
Source: Cancer Cell Int. 2026 May 10;26:249. doi: 10.1186/s12935-026-04266-7 (PMC13340015; doi:10.1186/s12935-026-04266-7)
Supplement: Supplementary file 1 — Supplementary Material 1 [file 12935_2026_4266_MOESM1_ESM.pdf]

Supplementary Materials—Original Uncropped Blots

Uncropped blots for Figure 1F

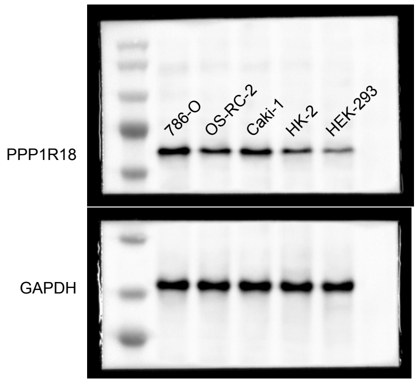

Uncropped blots for Figure 3A

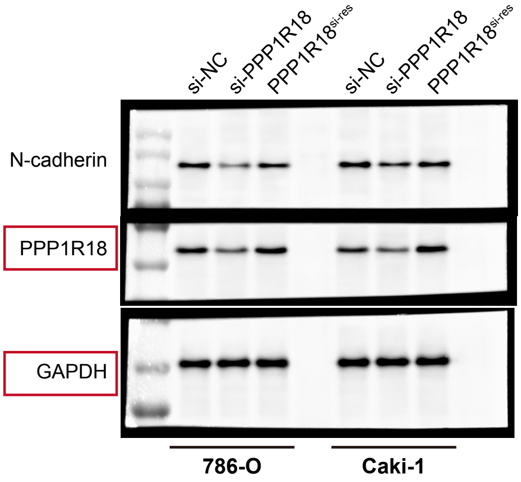

Uncropped blots for Figure 1G

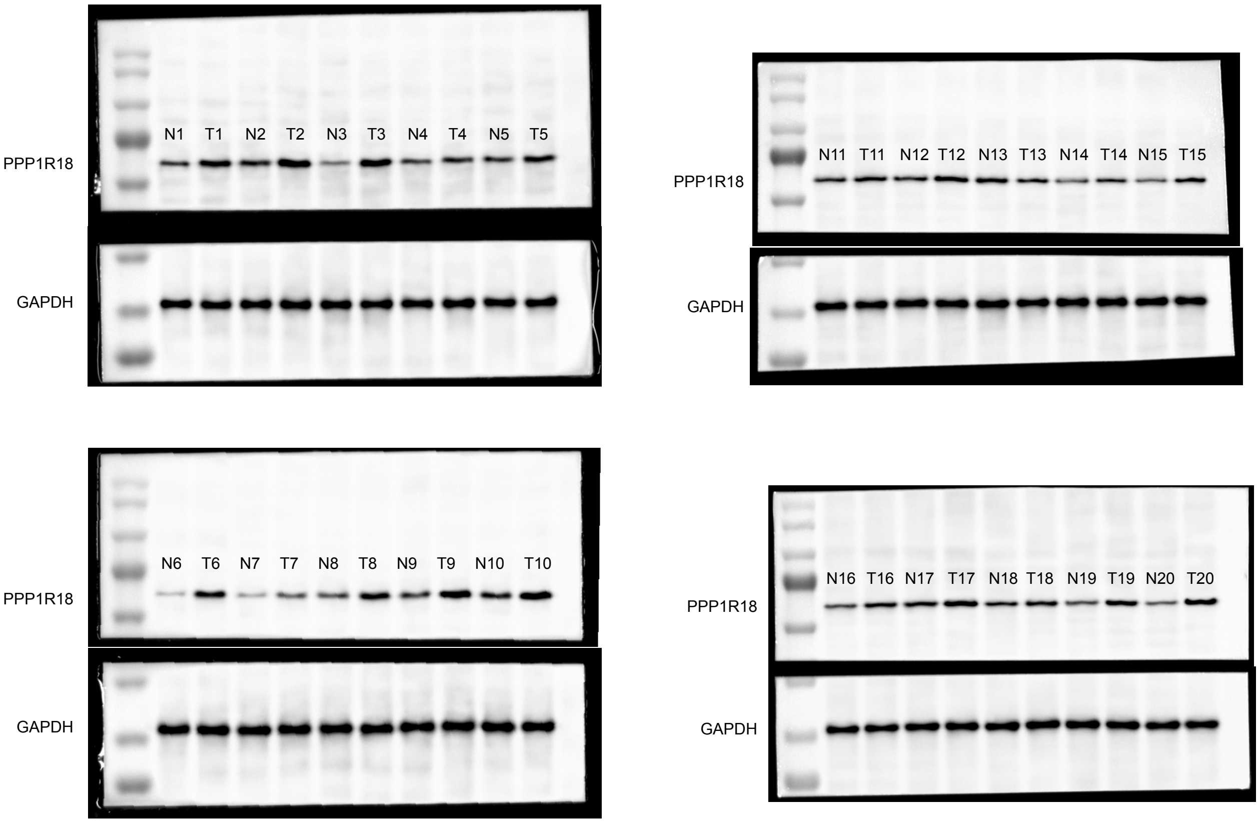

Uncropped blots for Figure 4D

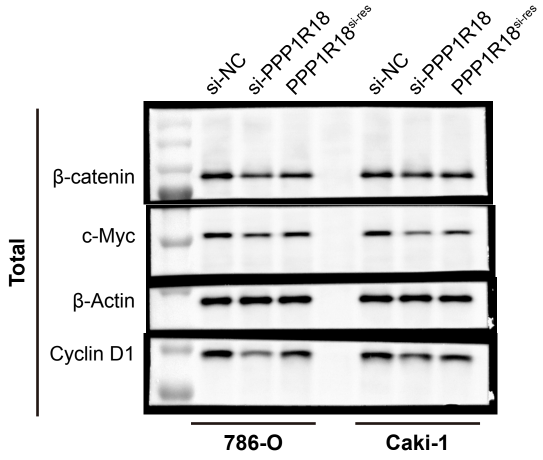

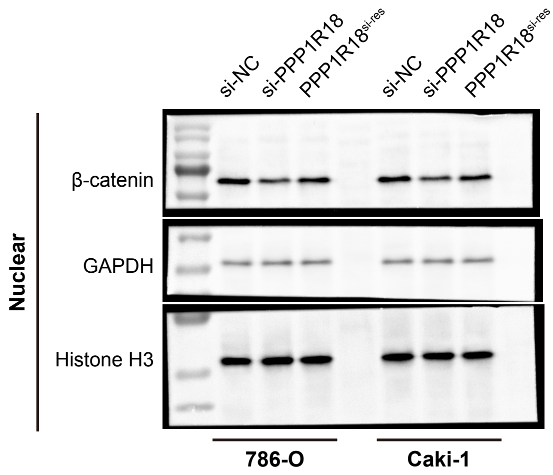

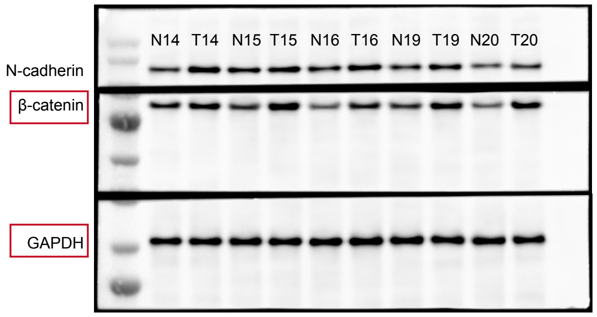

Uncropped blots for Figure 4E

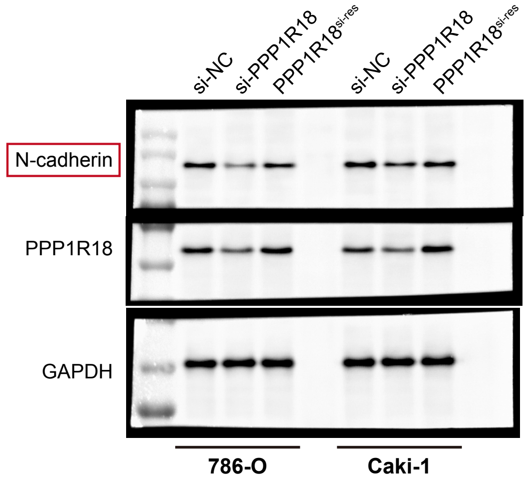

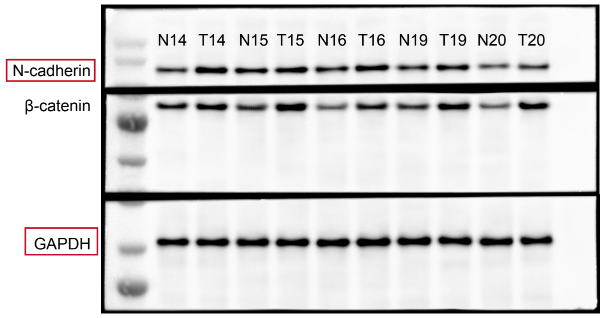

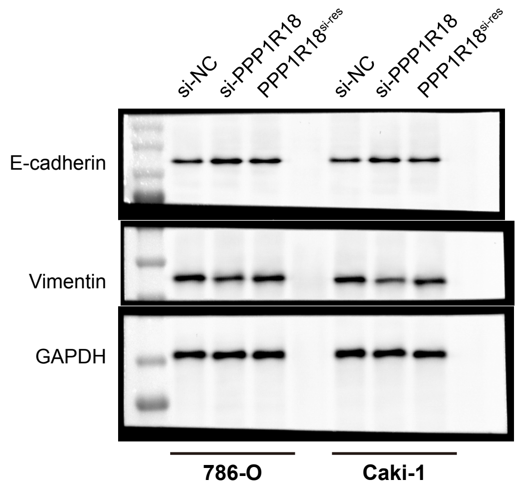

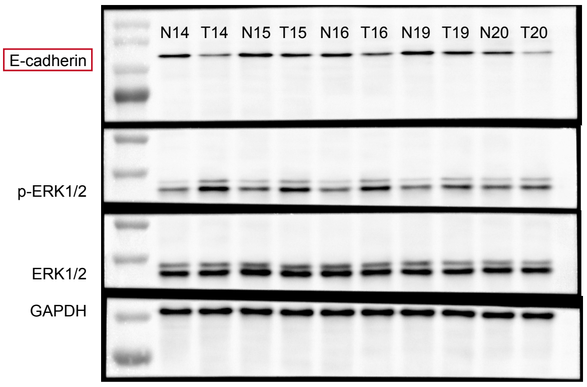

*The blots shown for p-ERK and total ERK were obtained from the same membrane. The membrane was stripped and re-probed sequentially with the indicated antibodies.

Uncropped blots for Figure 5A

*The blots shown for p-ERK and total ERK were obtained from the same membrane.
The membrane was stripped and re-probed sequentially with the indicated antibodies.

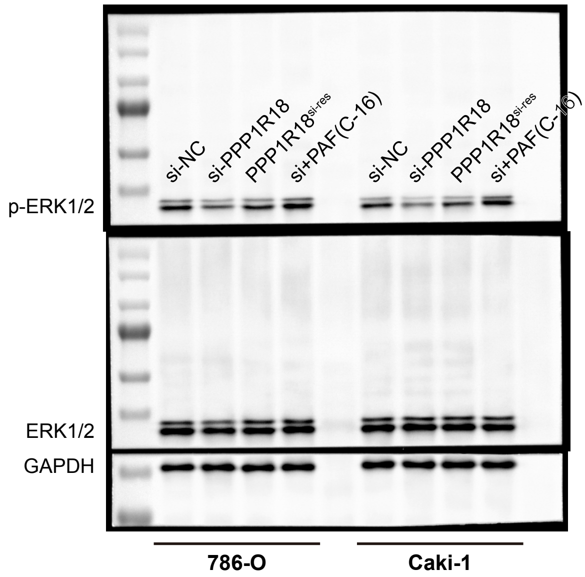

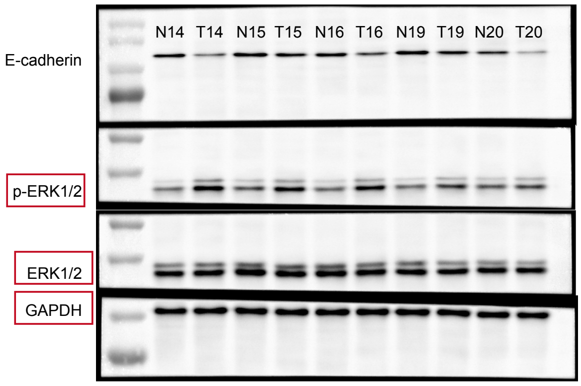

*The blots shown for p-ERK and total ERK were obtained from the same membrane.
The membrane was stripped and re-probed sequentially with the indicated antibodies.

Uncropped blots for Figure 5B

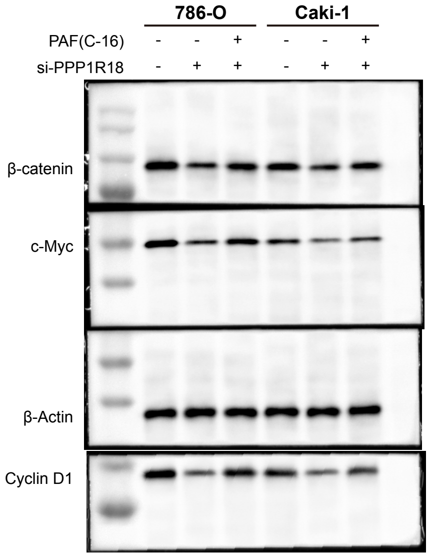

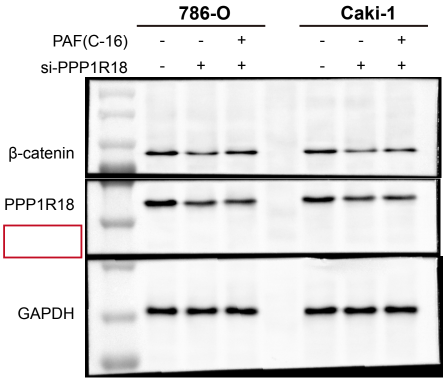

*The blots shown for β-Actin and c-Myc were obtained from the same membrane.
The membrane was stripped and re-probed sequentially with the indicated antibodies.

Uncropped blots for Figure 6D

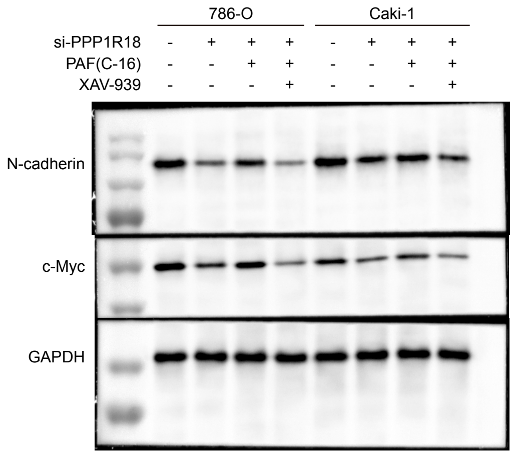

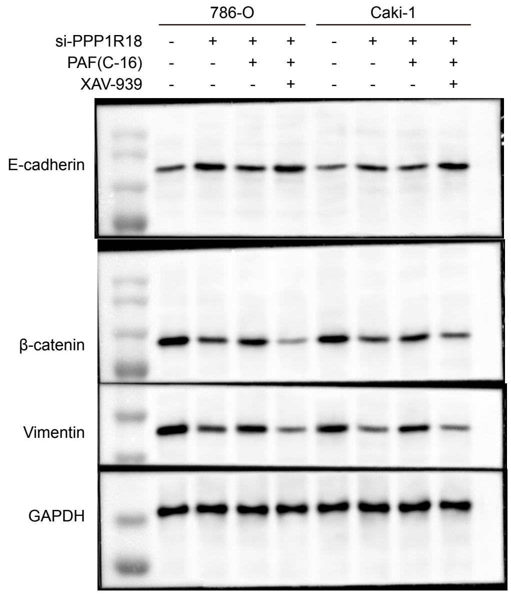

*The blots shown for β-catenin and E-cadherin were obtained from the same membrane. The membrane was stripped and re-probed sequentially with the indicated antibodies.

Uncropped blots for Figure 7E

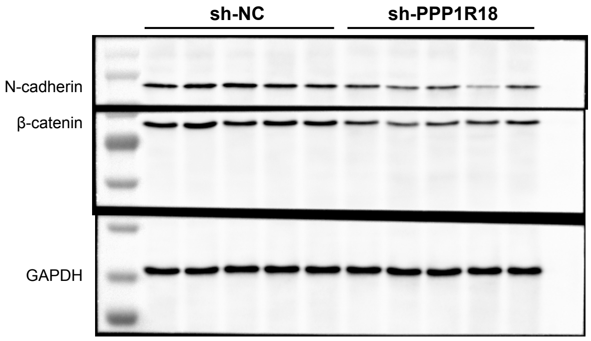

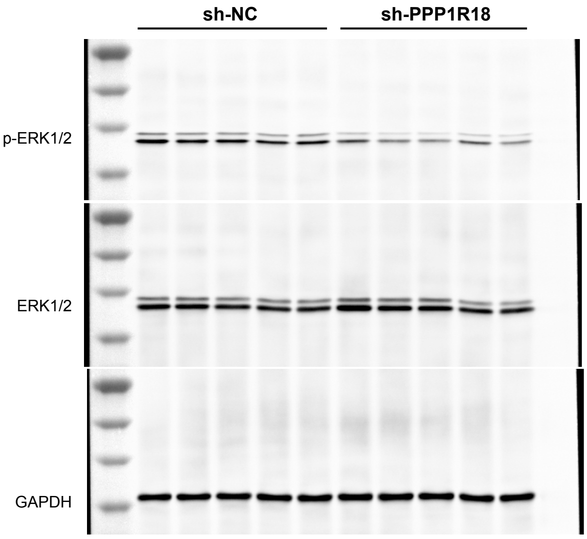

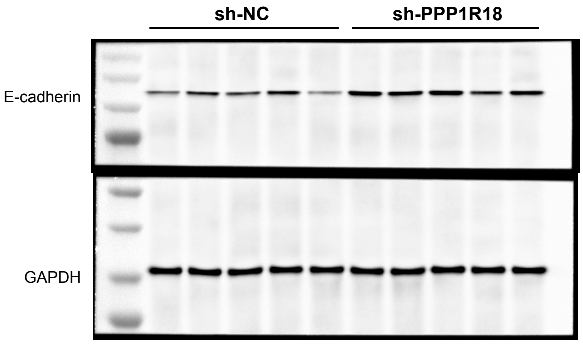

*The blots shown for p-ERK, ERK and GAPDH were obtained from the same membrane. The membrane was stripped and re-probed sequentially with the indicated antibodies.
